# MaxEnt model strategies to studying current and future potential land suitability dynamics of wheat, soybean and rice cultivation under climatic change scenarios in East Asia

**DOI:** 10.1371/journal.pone.0296182

**Published:** 2023-12-21

**Authors:** Shahzad Ali, Tyan Alice Makanda, Muhammad Umair, Jian Ni

**Affiliations:** 1 College of Life Science, Zhejiang Normal University, Jinhua, China; 2 Department of Agriculture, Hazara University, Mansehra, Pakistan; University of Uyo, NIGERIA

## Abstract

Climate change and variability are projected to alter the geographic suitability of lands for crops cultivation. Accurately predicting changes in the potential current and future land suitability distribution dynamics of wheat (*Triticum aestivum*), soybean (*Glycine max*) and rice (*Oryza sativa*) crops due to climate change scenarios is critical to adapting and mitigating the impacts of bioclimatic changes, and plays a significant role in securing food security in East Asia region. This study compiled large datasets of wheat, soybean and rice occurrence locations from GBIF and 19 bioclimatic variables obtained from the WorldClim database that affect crops growth. We recognized potential future suitable distribution regions for crops under the one socioeconomic pathway, (SSP585) for 2021–2040 and 2041–2060, using the MaxEnt model. The accuracy of the MaxEnt was highly significant with mean AUC values ranging from 0.833 to 0.882 for all models evaluated. The jackknife test revealed that for wheat, Bio4 and Bio12 contributed 17.6% and 12.6%, for soybean Bio10 and Bio12 contributed 15.6% and 49.5%, while for rice Bio12 and Bio14 contributed 12.9% and 36.0% to the MaxEnt model. In addition, cultivation aptitude for wheat, soybean, and rice increased in southeast China, North Korea, South Korea, and Japan, while decreasing in Mongolia and northwest China. Climate change is expected to increase the high land suitability for wheat, soybean, and rice in East Asia. Simulation results indicate an average decrease of unsuitable areas of -98.5%, -41.2% and -36.3% for wheat, soybean and rice from 2060 than that of current land suitability. In contrast, the high land suitable for wheat, soybean and rice cultivation is projected to increase by 75.1%, 68.5% and 81.9% from 2060 as compared with current. The findings of this study are of utmost importance in the East Asia region as they present an opportunity for policy makers to develop appropriate adaptation and mitigation strategies required to sustain crops distribution under future climates. Although the risks of wheat, soybean and rice cultivation may be significantly higher in the future because of high temperatures, heat waves, and droughts caused by climate change.

## 1. Introduction

As a result of global warming, crop yield reduction is becoming significantly common in these days [1]. According to the Intergovernmental Panel on Climate Change (IPCC) report global mean temperatures are projected to rise by 0.3 to 0.5°C by 2100, than that of mean temperature between 1986 and 2005 [2, 3]. Crops production affected by climate change, which has not only implications for farmer incomes, but also for global food security [4, 5]. Climate change has caused ecological changes, including changes in phonological events, shifts in the ranges of many species, the invasion of non-native species, and fluctuations in grain yield production [6–8], especially with regard to agricultural production, which is highly dependent on specific climatic and environmental conditions. Climate change alters the current land suitability for certain crops and affects crop growth and production [8, 9]. To accurately assess the impacts of climate change on crop production, predicting the potential global distribution of crops is a critical issue [10]. However, there is relatively little research on the effect of climate change on the global distribution of crops. Land suitability is one of the factors that affect the potential distribution of a particular crop. Under the climate change scenarios the most important steps in ensuring food securities are the quantitative analysis of ecological factors and a comprehensive assessment of the potential distribution of specific crops [11].

Suitability is often considered among crop requirements and land characteristics [12]. For land suitability assessment the climatic conditions, soil quality, and geographic characteristics of a given region are the most significant parameters [13]. Species distribution models (SDMs) have been usually used to predict potential distributions of various crops [14]. SDM involves collecting species occurrence data, correlating these events with bioclimatic variables and producing maps that forecast past, present and future crops distributions [15]. Based on occurrence data and taking into account the environmental variables that affect the target species and a relationship between them can be assessed through statistical algorithms built into SDM [16]. MaxEnt is currently the most commonly used model [15, 17]. MaxEnt predictions have the highest entropy and are closest to geographic uniformity, ensuring the accurate predictions [1, 18]. MaxEnt has revealed excellent prediction performance in relative studies of several modeling approaches [19, 20].

The potential area for rice cultivation is determined by land suitability [9, 21]. Earlier research works have to studied the land suitability of rice based on climatic parameters; however, the selection of those bioclimatic variables is mostly based upon the expertise [22, 23]. Furthermore, there are four types of climatic variables that affect the land suitability of rice such as cumulative temperature, insolation, temperature, and rainfall [24, 25]. Since it is difficult to increase the potential yield and closes the yield gap due to the rapid decrease in arable land, further increasing the cropland harvesting frequency (CHF) is considered an actual approach to improve future rice cultivation land [8, 26]. However, potential planting areas in East Asia suitable for rice production are still unknown.

Wheat, the 3^rd^ main crop on the world which played a significant part in sustaining food security [27, 28]. The MaxEnt model has been effectively used by Balkovič et al. [28] to recognize areas suitable for wheat cultivation. Also, this study applied the MaxEnt to evaluate the influence of bioclimatic change on the geographic distribution of selected wheat producing regions. This study evaluated wheat current and future crop suitability using only existing data. For wheat with a huge environmental range and uneven spatial distribution, selecting a representative sample from big data remains an urgent problem. According to statistics released by the FAO, soybean is the 4^th^largest crop in the world, with about 120 million hectares cultivated in 2016. It is also a major source of protein, feed, and cooking oil. Soybeans have become one of the world’s most significant agricultural products and play a key role in securing the world’s food supply [29]. Cold regions provide the greatest advantage for expanding the planting area of soybean, accounting for about half of the total soybean production area of China [30]. Thus, a balance of current and future soybean aptitude and appropriate distribution coverage is required. In this study, we attempt to address this issue with the rarely used MaxEnt model to study soybean potential distribution and determine their potential future distribution. The goal is to optimize the soybean planting layout in areas of limited land resources and efficiently demonstrate the social, economic and ecological benefits of soybean cultivation.

Global bioclimatic changes are probable to have a major impact on future wheat, soybean and rice cultivation. In order to meet the growing global demand, finding potential current and future suitable wheat, soybean, and rice planting areas has become an urgent problem to meet the global food security [5]. Therefore, the purposes of this study are to: (1) identify main environmental factors affecting the geographic range and the land suitability of wheat, soybean and rice; (2) which regions are more suitable for potential crops cultivation of wheat, soybean and rice under present and future bioclimatic scenarios; (3) to predict the land suitability and potential distribution of wheat, soybean and rice in the future (2021–2060) dynamics of bioclimatic changes. This information will be useful for policy makers to decide the magnitude of area expansion and reduction for these crops in order to maintain regional food security in the future.

## 2. Materials and methods

### 2.1. Study region

East Asia region contains Mongolia, China, North Korea, South Korea, and Japan, from 5°N to 55°N, and 70°E to 140°E. The study region is located in the northeastern part of the Asian continent, covering an area of about 5,125,000 km^2^. The region includes a variety of climatic zones, including tropical, subtropical, temperate, boreal, humid, semi-arid and arid regions. The average annual precipitation is 256 mm; more than 56% fall in summer and less than 4% in winter. The average temperature in summer reaches above 17°C, and the average temperature in winter drops below -7°C respectively [31].

### 2.2. Species occurrence record

**Fig 1:**
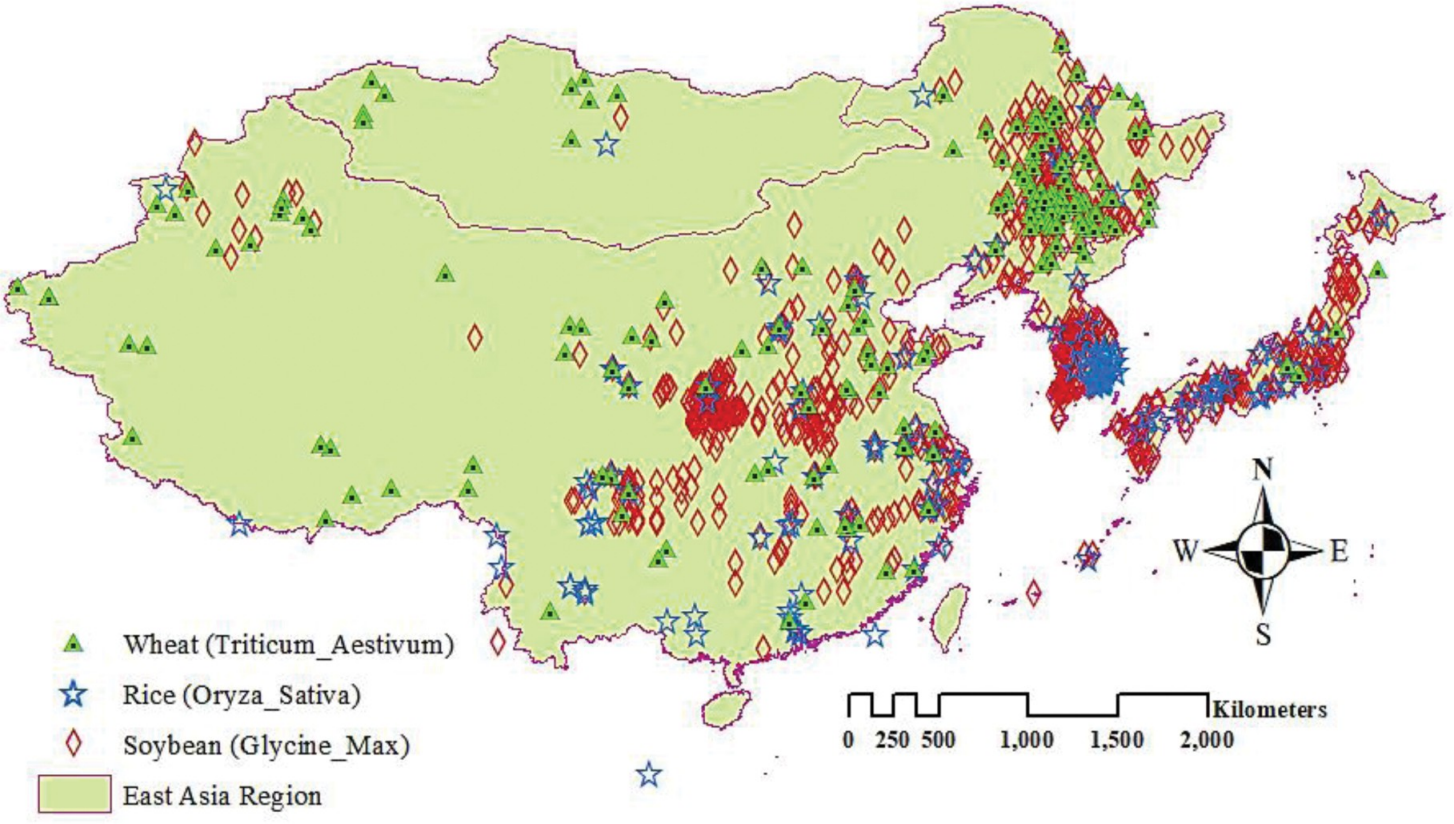
Occurrence points of wheat, rice and soybean in East Asia region. Occurrence points Data were accessed from GBIF.org (https://www.gbif.org/occurrence/search) database. GBIF = Global Biodiversity Information Facility.

**Fig 2:**
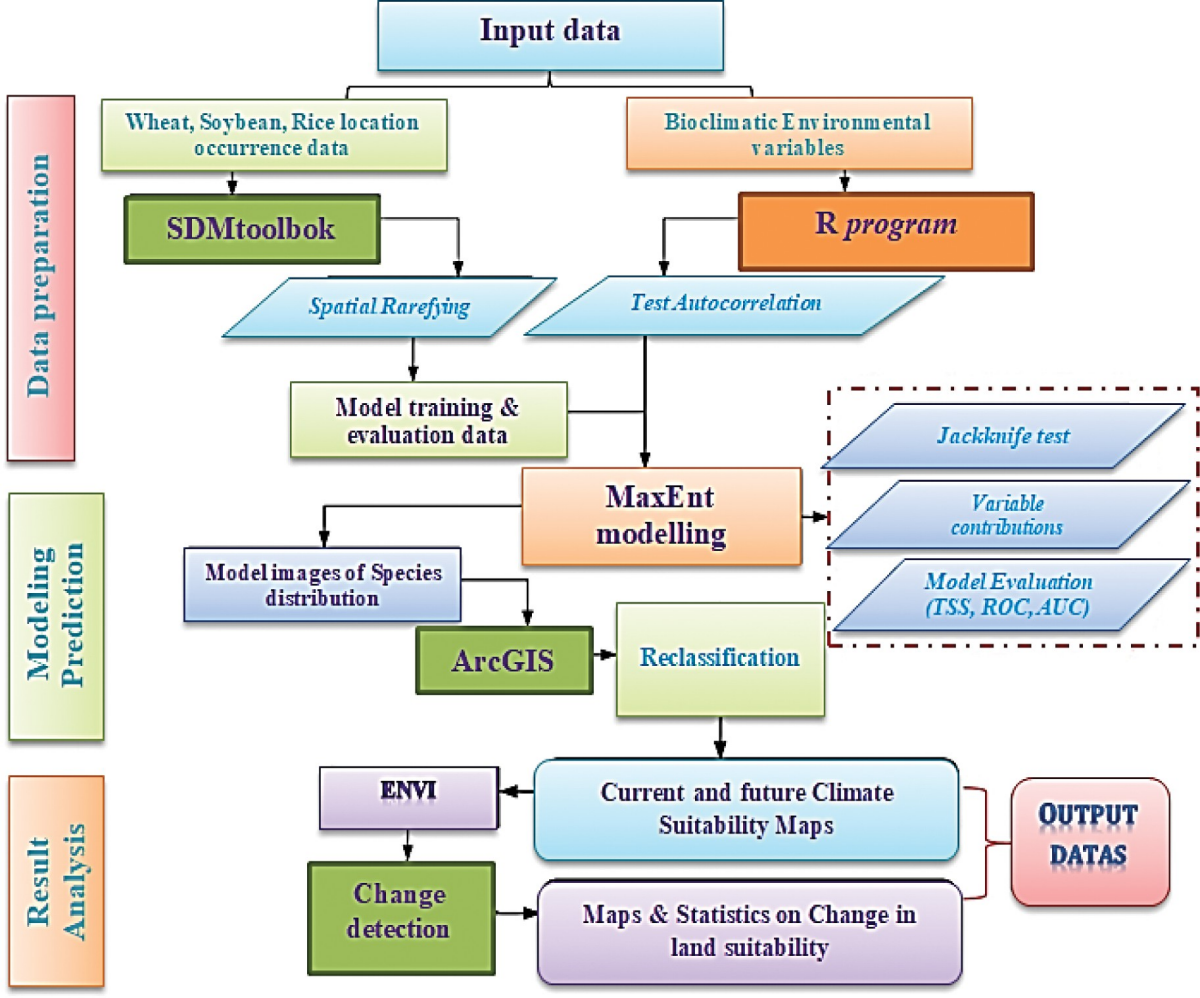
Flowchart summarizing the processing methodology used in this study.

In this study, we estimated distribution models using public databases collecting current distribution data for wheat, soybean, and rice. Global distribution data for wheat, soybean and rice were collected from the Global Biodiversity Information Facility database [32]. In addition, we subsequently reviewed this data set critically from records on the GBIF database and manually removed unreliable and ambiguous records through the “Description of Occurrence” column for unconfirmed species identification. After that we excluded duplicate records and those whose geographical location was not precisely defined (uncertainty in meters > 10,000 m) for more reliable assessment. After this selection, we obtained a total of 696 presence records of soybean, 193 presence records of wheat, and 155 presence records of rice. In Fig 1 show the general distribution of crops occurrence record. The occurrence records were used to produce present distribution models for wheat, soybean, and rice species. Fig 2 show a flowchart and processing methodology of study.

### 2.3. Current bioclimatic variables

For the SDM of wheat, soybean, and rice, we initially considered 19 bioclimatic variables (Bio1-Bio19), were obtained as a raster layers from Worldclim 2.1 database (https://www.worldclim.org/data/worldclim21.html) available at bio 30 arc-seconds with 1 km^2^ spatial resolution at the equator (Table 1). Bioclimatic variables derived from monthly rainfall and temperature that define seasonal and yearly trends, which are important than the existence as a species in a place. These bioclimatic variables have been used in environmental studies to assess the influence on possible species distribution [33]. All analyzes, calculations, and transformations were performed in ArcGIS 10.7.1 Esri. To identify highly correlated variables (|r| > 0.74) and minimize the effects of multi-collinearity and model overfitting, the person correlation coefficient method was used [34].

**Table 1:** Bioclimatic environmental variables used in MaxEnt model.

Variable code	description	Unit	Source
Bio1	Annual mean temperature	°C	
Bio2	Mean diurnal range	°C	
Bio3	Isothermality	°C	
Bio4	Temperature seasonality	°C	
Bio5	Maximum temperature of the warmest month	°C	
Bio6	Minimum temperature of the coldest month	°C	
Bio7	Temperature annual range	°C	
Bio8	Mean temperature of the wettest quarter	°C	
Bio9	Mean temperature of the driest quarter	°C	WorldClim^a^^,^^b^
Bio10	Mean temperature of the warmest quarter	°C	
Bio11	Mean temperature of the coldest quarter	°C	
Bio12	Annual precipitation	mm	
Bio13	Precipitation of the wettest month	mm	
Bio14	Precipitation of the driest month	mm	
Bio15	Precipitation seasonality	mm	
Bio16	Precipitation of the wettest quarter	mm	
Bio17	Precipitation of the driest quarter	mm	
Bio18	Precipitation of the warmest quarter	mm	
Bio19	Precipitation of the coldest quarter	mm	

^a^Data for current climate conditions were accessed from WorldClim

(https://www.worldclim.org/data/worldclim21.html)

^b^Data for future climate projections were accessed from WorldClim

(https://www.worldclim.org/data/cmip6/cmip6_clim30s.html)

2.4. Future climate change scenarios

We modeled the future distributions of wheat, soybean, and rice to examine differences in their potential habitats under different climate scenarios. The shared socioeconomic pathway (SSP) consists of five main ACCESS-CM2-SPPs (SSP119, SSP126, SSP245, SSP370, and SSP585). Among them, we use the intermediate shared SSP (SSP585) for two steps: 2021–2040 and 2041–2060. The SSPs scenarios belong to the "SSPs socioeconomic family", which stands for "sustainability" [35]. Bioclimatic data for these climatic model scenarios were downloaded from the Worldclim 2.1 database (https://www.worldclim.org/data/cmip6/cmip6_clim30s.html).

### 2.5. MaxEnt model description

The MaxEnt model is a general-purpose machine learning model based on a precise and straight forward mathematical formulation [36, 37]. It has also been defined as a presence only model that uses predictive data sets to discriminate crops occurrence record [14, 36]. The model utilises categorical and continuous datasets [37, 38]. Although the underlying prediction of those areas has been systematically sampled from most existing lands, MaxEnt model is often constructed from spatially base occurrence records [35]. The model offers both a userfriendly graphical user interface and command-line functions. MaxEnt is one of the most popular niche-based methods for modeling geographical crops distribution [37]. The model also provides valuable tools such as jackknife tests, species environment curve, and area under the receiver operating characteristic curve AUC and ROC [36]. In this research MaxEnt model version 3.4.4 (https://www.cs.princeton.edu/~schapire/ MaxEnt) was used to simulate the distribution of three crops (wheat, soybean and rice) in East Asia region.

### 2.6. Validation and application of MaxEnt model

The Receiver operating characteristic (ROC) curves were used to validate the performance with the MaxEnt model. The ROC curves are a standard way to evaluate the MaxEnt model predictive accuracy [39]. The area of the ROC curve is a threshold independent measure of model performance, called (AUC) area under the ROC curve [40]. The AUC values greater than 0.9 show very high accuracy, values 0.7–0.9 show high accuracy, and values less than 0.7 show low accuracy [41]. The MaxEnt model to predict short-term (2021–2040) and long-term (2041–2060) land suitability for soybean, wheat, and rice distribution under different future bioclimatic condition. Habitat suitability for wheat, soybean, and rice was divided into 4 grades according to possible explanations from the IPCC [42] 8, and previous study [43], 0.0–0.05 is not suitable; 0.05–0.33 low suitable; 0.33–0.66 moderate suitable; 0.66–1.00 high suitable. The robustness of the model result was checked, the regularization multiplier was adjusted to 3 and the maximum number of background points was also adjusted to 10000. We replicated our model 10 times and used the average of the 10 probability outputs to determine the optimum habitat suitability and performance of the models. This also helps to evaluate uncertainty in the model. The output is in ASCII format, and ArcGIS 10.7.1 Esri was used for final map processing and visualization. To evaluate bioclimatic variables and determine a crops potential distribution, we use jackknife tests [44]. We also make crops response curves to assess the correlation between the land suitability and bioclimatic variables.

### 2.7. Pearson correlation analysis

Tables 2–4 present the Pearson correlation analyzes for wheat, soybean, and rice. In our study, we used bioclimatic variables based on rainfall and temperature [33]. For this reason, bioclimatic variables are highly correlated with each other. Distribution modeling using highly correlated bioclimatic data can affect the forecasting process and overestimate distributions [45]. To identify and exclude highly correlated variables (|r| > 0.74) and minimize the effects of model overfitting, the Pearson correlation coefficient method was used. For the 19 variables with correlation coefficient values >0.74 or < − 0.74, the Pearson correlation analysis of wheat, soybean, and rice showed strong correlations between paired variables (Tables 2–4). Furthermore, a jackknife test constructed throughout the MaxEnt model showed that these variables had a small effect on species distribution.

**Table 2:** Wheat correlation analysis results of the bioclimatic environmental variables.

	Bio1	Bio2	Bio3	Bio4	Bio5	Bio6	Bio7	Bio8	Bio9	Bio10	Bio11	Bio12	Bio13	Bio14	Bio15	Bio16	Bio17	Bio18
**Bio1**																		
**Bio2**	0.5468																	
**Bio3**	-0.5669	-0.5347																
**Bio4**	0.3746	0.3632	**-0.9536**															
**Bio5**	-0.5687	-0.7454	**0.7719**	-0.6099														
**Bio6**	**-0.8916**	-0.2116	0.3546	-0.1478	0.3272													
**Bio7**	-0.5943	-0.4308	-0.2305	0.4101	0.1056	0.4958												
**Bio8**	-0.3995	-0.1475	0.2747	-0.3804	-0.1483	0.1854	0.2405											
**Bio9**	**0.9226**	0.3802	-0.4423	0.2061	-0.4723	**-0.9880**	-0.5086	-0.1286										
**Bio10**	0.1223	0.4492	0.0060	0.0654	-0.0712	0.2708	-0.4698	-0.6093	-0.2383									
**Bio11**	**-0.8158**	-0.3269	0.0290	0.1408	0.0701	**0.7955**	**0.8593**	0.4811	**-0.7691**	-0.2500								
**Bio12**	0.0580	0.2465	0.4812	-0.7113	-0.0132	-0.1991	-0.5511	0.5650	0.2632	-0.1916	-0.3065							
**Bio13**	-0.2857	-0.1792	-0.3142	0.5712	0.0135	0.5137	0.5241	-0.4454	-0.5490	0.4078	0.4842	**-0.9168**						
**Bio14**	-0.5039	-0.1266	0.6223	**-0.7419**	0.2779	0.2658	0.0117	**0.7717**	-0.2243	-0.4340	0.2580	**0.7981**	-0.6652					
**Bio15**	**0.9372**	0.4699	-0.3053	0.109	-0.4700	**-0.8350**	**-0.7925**	-0.3293	**0.8471**	0.2455	**-0.8805**	0.2048	-0.3503	-0.4141				
**Bio16**	0.0957	-0.0933	-0.7390	**0.8615**	-0.1992	-0.0628	0.7007	-0.3295	0.0488	-0.2535	0.3086	**-0.8083**	0.5878	-0.5879	-0.2021			
**Bio17**	-0.2507	-0.6904	0.7399	-0.6652	**0.9228**	-0.0470	-0.1568	-0.1815	-0.1110	-0.1568	-0.2659	0.1444	-0.2424	0.2400	-0.1343	-0.2888		
**Bio18**	-0.4279	-0.4201	-0.2529	0.5090	0.0625	0.5150	0.7378	-0.1644	-0.5700	0.0654	0.6705	**-0.8801**	**0.9278**	-0.4996	-0.5019	0.6461	-0.1932	
**Bio19**	0.4161	0.3195	**-0.9591**	**0.9654**	-0.7102	-0.2460	0.4096	-0.1882	0.3055	-0.0959	0.1552	-0.6201	0.4658	-0.6524	0.1715	**0.8205**	-0.7208	0.4753

Bio: Bioclimatic environmental variable (Bio1 ~ Bio19)

Bold: Strongly correlating (|r| > 0.74, p < 0.05)

N (replication number): 10

**Table 3:** Soybean correlation analysis results of the bioclimatic environmental variables.

	Bio1	Bio2	Bio3	Bio4	Bio5	Bio6	Bio7	Bio8	Bio9	Bio10	Bio11	Bio12	Bio13	Bio14	Bio15	Bio16	Bio17	Bio18
**Bio1**																		
**Bio2**	-0.7205																	
**Bio3**	0.3936	0.0535																
**Bio4**	-0.4170	0.1505	-0.4845															
**Bio5**	0.2280	-0.2948	-0.7150	0.0700														
**Bio6**	**0.7621**	-0.3247	0.5725	-0.6786	0.1279													
**Bio7**	**-0.7866**	0.3977	-0.7112	0.2229	0.1734	**-0.7798**												
**Bio8**	**0.8909**	**-0.8005**	0.2122	0.0138	0.2103	0.4670	-0.7405											
**Bio9**	-0.1088	-0.2082	-0.1249	-0.4904	-0.1094	-0.2384	0.5173	-0.2575										
**Bio10**	-0.6668	0.0300	-0.7003	0.2823	0.0876	**-0.7913**	**0.8832**	-0.5138	0.6062									
**Bio11**	-0.3177	0.3823	-0.3336	-0.4868	0.3459	-0.0546	0.6396	-0.6309	0.5311	0.3434								
**Bio12**	**0.8799**	-0.4258	0.5625	-0.4383	0.141	**0.9354**	**-0.915**	0.7074	-0.42	**-0.8901**	-0.3207							
**Bio13**	-0.4327	0.7034	-0.2622	0.5798	0.0989	-0.4614	0.3018	-0.3078	-0.4523	-0.0458	0.1068	-0.3268						
**Bio14**	**0.7597**	-0.3321	0.1794	-0.0747	0.3058	0.3824	-0.5093	0.731	-0.2309	-0.6134	-0.1836	0.6017	0.2165					
**Bio15**	0.4620	-0.6891	-0.2244	0.1700	0.1983	-0.2087	-0.0351	0.6515	0.3333	0.1717	-0.2723	0.0062	-0.1334	0.5667				
**Bio16**	-0.5850	0.4956	-0.1494	**0.8701**	-0.345	-0.7139	0.2292	-0.235	-0.4309	0.1877	-0.4220	-0.5202	0.7017	-0.1626	-0.0421			
**Bio17**	-0.0512	-0.1365	-0.1961	**0.8069**	0.0754	-0.1774	-0.276	0.3274	-0.7469	-0.0892	**-0.7709**	0.0551	0.2037	-0.0480	0.0189	-0.5428		
**Bio18**	-0.1677	-0.0139	-0.4132	-0.3345	0.5421	0.2449	0.2926	-0.3664	0.1318	0.2620	0.5654	-0.0177	-0.3356	-0.4822	-0.4865	-0.5428	0.7523	
**Bio19**	-0.5256	-0.0526	-0.6336	**0.7631**	-0.0043	**-0.9127**	0.5967	-0.1380	0.1813	**0.7739**	-0.2091	**-0.7783**	0.2363	-0.2949	0.4386	0.6347	0.4045	-0.2441

Bio: Bioclimatic environmental variable (Bio1 ~ Bio19)

Bold: Strongly correlating (|r| > 0.74, p < 0.05)

N (replication number): 10

**Table 4:** Rice correlation analysis results of the bioclimatic environmental variables.

	Bio1	Bio2	Bio3	Bio4	Bio5	Bio6	Bio7	Bio8	Bio9	Bio10	Bio11	Bio12	Bio13	Bio14	Bio15	Bio16	Bio17	Bio18
**Bio1**																		
**Bio2**	0.0326																	
**Bio3**	-0.4637	**-0.7873**																
**Bio4**	-0.3524	-0.4246	**0.8034**															
**Bio5**	**0.8044**	0.0532	-0.6312	-0.7035														
**Bio6**	-0.3698	0.2006	0.0978	0.2741	-0.6048													
**Bio7**	-0.5234	-0.5784	0.6298	0.5873	-0.3897	0.1971												
**Bio8**	**-0.7811**	-0.2974	0.3850	-0.0505	-0.4418	0.2548	0.4261											
**Bio9**	-0.7144	0.1027	0.0680	-0.3087	-0.3384	0.0351	-0.0153	0.8426										
**Bio10**	-0.5160	0.4919	0.1148	0.5346	-0.7101	0.3552	0.0792	-0.1111	0.0576									
**Bio11**	-0.5982	-0.1196	0.4528	0.1873	-0.4894	-0.2865	-0.0369	0.4724	0.697	0.3153								
**Bio12**	0.1336	-0.4877	0.2826	0.3789	0.0237	0.3729	0.6786	-0.0524	-0.5669	-0.2516	-0.6977							
**Bio13**	-0.1620	0.0733	0.0997	-0.1913	-0.1535	-0.3325	-0.5631	0.2268	0.5905	0.0527	**0.8210**	**-0.8765**						
**Bio14**	0.3876	0.5945	-0.5024	-0.1136	0.3126	-0.4856	-0.5051	**-0.7514**	-0.3329	0.4021	0.0218	-0.4787	0.1389					
**Bio15**	0.2254	-0.4097	0.4234	0.1662	-0.1397	0.0063	-0.3688	-0.0095	-0.0183	-0.2547	0.2995	-0.1837	0.5792	-0.2586				
**Bio16**	-0.2458	**0.7764**	-0.3337	0.147	-0.4548	0.6713	-0.1615	-0.1996	-0.0534	**0.7811**	-0.1771	-0.0895	-0.1922	0.2926	-0.3280			
**Bio17**	-0.2860	-0.4874	0.2385	-0.1363	-0.0396	0.3681	0.5236	0.7183	0.3118	-0.5448	-0.2170	0.5414	-0.3256	**-0.9249**	-0.0389	-0.3319		
**Bio18**	0.3898	**-0.7985**	0.5762	0.3905	0.1987	-0.5318	0.1646	-0.2528	-0.4301	-0.4033	0.1091	0.2293	0.0968	-0.0454	0.5431	**-0.7449**	-0.0814	
**Bio19**	0.1016	0.4211	-0.4364	-0.6157	0.2976	-0.5483	**-0.7516**	-0.0058	0.4960	-0.0574	0.5369	**-0.9413**	**0.8168**	0.4681	0.1689	-0.1273	-0.4429	-0.1394

Bio: Bioclimatic environmental variable (Bio1 ~ Bio19)

Bold: Strongly correlating (|r| > 0.74, p < 0.05)

N (replication number): 10

## 3. Results and discussion

### 3.1. MaxEnt model evaluation and Jackknife tests of bioclimatic variables

**Fig 3:**
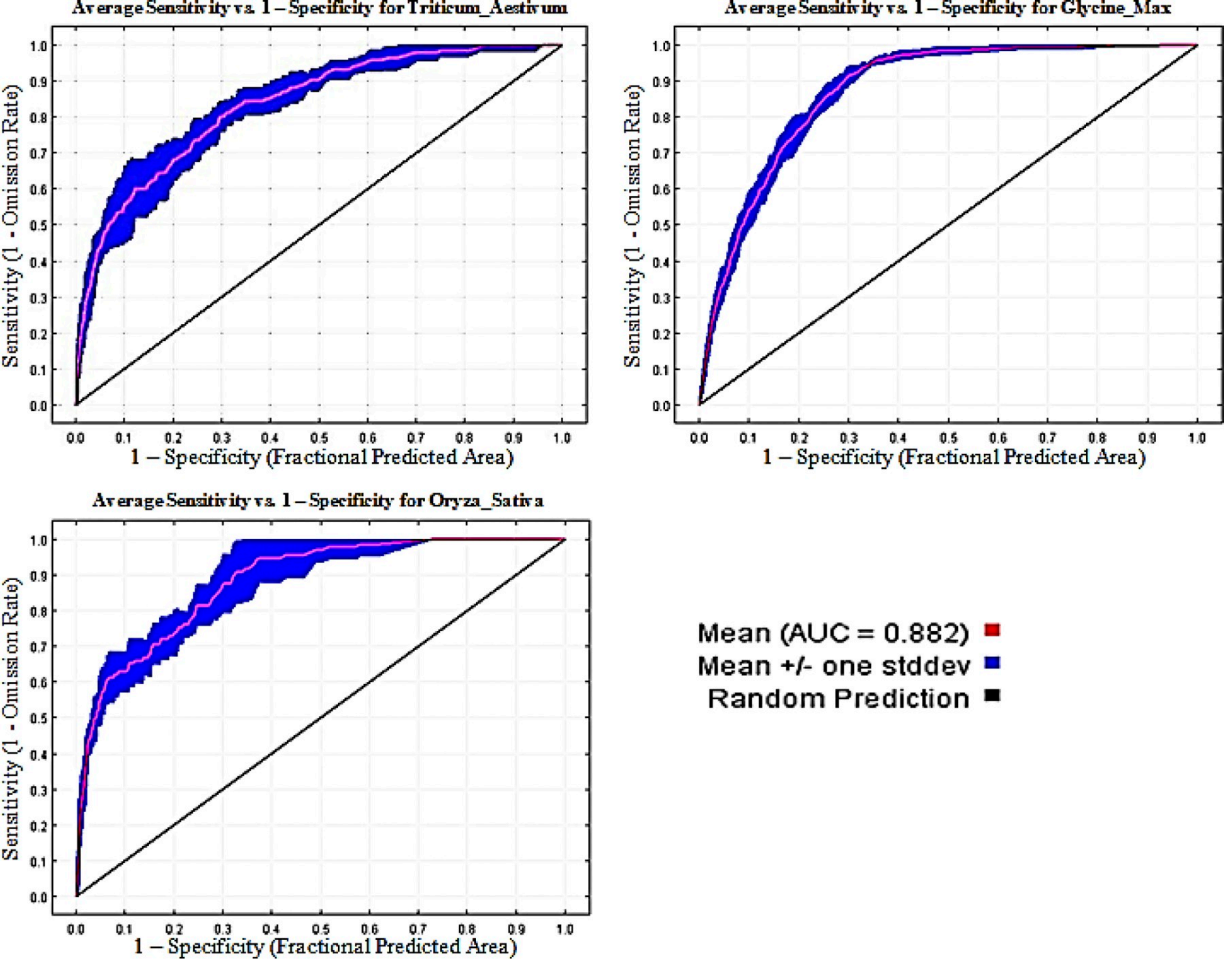
Receiver operating characteristic (ROC) curve and AUC value under the current (1970–2000) period (10 replicated runs).

This study we use a MaxEnt bioclimatic model to examine which bioclimatic variables better explain the distribution of wheat, soybean, and rice species. In this study, the performance of the MaxEnt model was examined in terms of ROC curves and AUC values (Fig 3). The AUC of the ROC curve in MaxEnt is generally regarded as an overall measure of model performance across all thresholds, prediction strengths and gave AUC values of 0.740, respectively [46, 47]. Earlier work has indicated that it is appropriate to link the performance between various training occurrence datasets [48]. The mean AUC values for wheat, soybean and rice of ten replicate were 0.83, 0.87 and 0.88, respectively, higher than 0.5 for the stochastic model, validating the good results for the simulations [49]. These values represent the average of repeated runs and are above 0.8, thus indicating that the MaxEnt model can satisfactorily estimate land suitability. ROC curve and AUC results showed that the MaxEnt model was very reliable and could accurately reflect the distribution of wheat, soybean and rice in East Asia. While, earlier research has shown that droughts will become more frequent and severe due to climate change [50, 51], which could significantly disturb wheat cultivation area [42].

**Fig 4:**
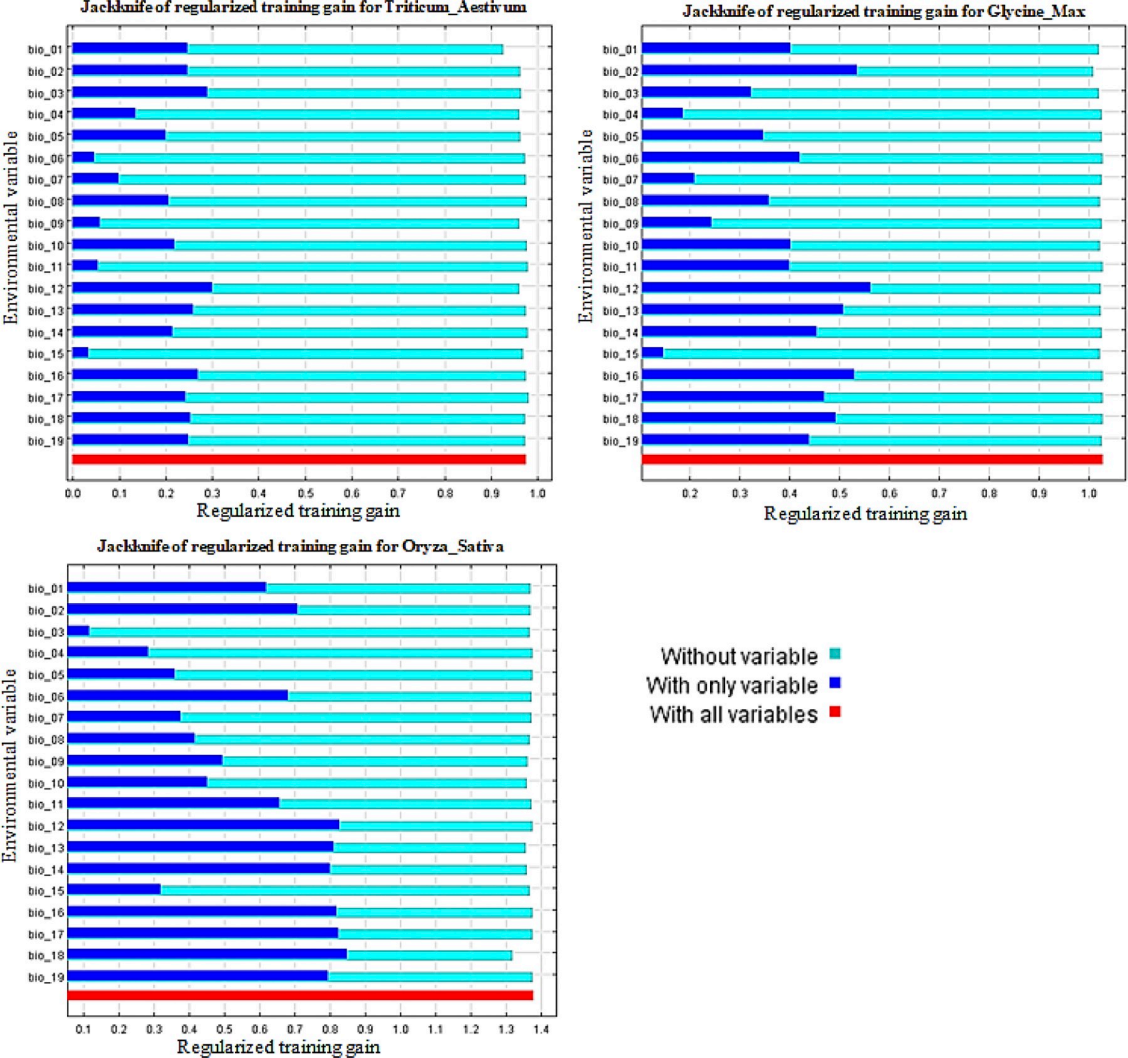
Results of Jackknife regularized training grain test of the MaxEnt model for evaluating the relative importance of bioclimatic environmental variables for wheat, soybean and rice occurrence.

**Fig 5:**
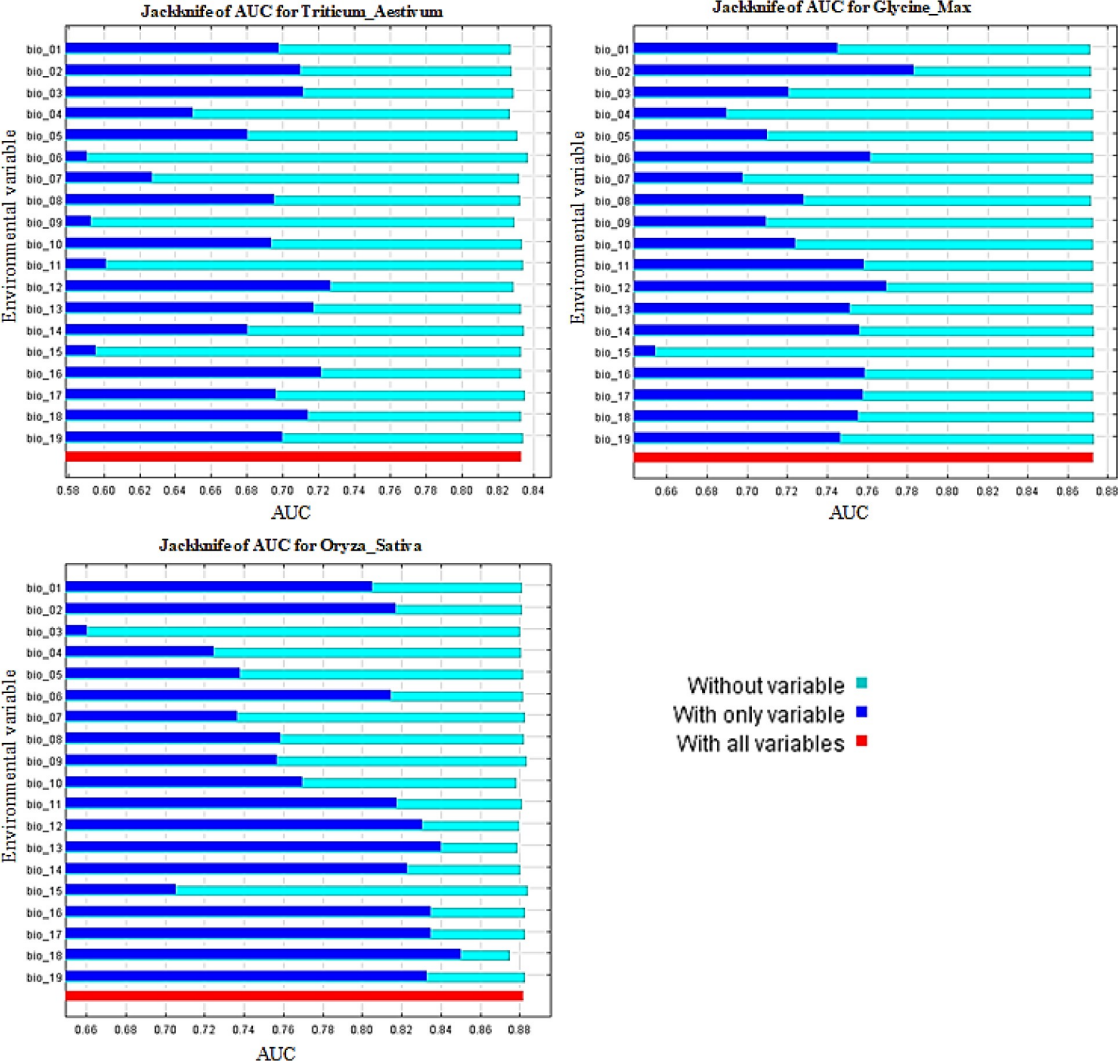
Results of Jackknife AUC test of the MaxEnt model for evaluating the relative importance of bioclimatic environmental variables for wheat, soybean and rice occurrence.

Climatic factors affecting regional suitability and rice productions of wheat and soybean were derived from 19 bioclimatic variables (Figs 4 and 5). The contribution of each of these predictors in affecting geographic suitability for crop cultivation was analyzed in a histogram of jackknife tests, one of the outputs from the MaxEnt model. Climate is one of the most important factors influencing the geographic distribution of plant species, vegetation patterns, and community structure [52]. Figs 4 and 5 show the jackknife regularization training to the grain test and the jackknife AUC test on the environment variable. When used alone, Bio1, Bio2, Bio3, Bio9, Bio12, Bio13 and Bio16 provided the highest wheat gain and AUC tested. Precipitation in the warmest season (Bio18) and precipitation in the coldest season (Bio19) also contributed more to the wheat model. When the sample size is greater than 30 the MaxEnt provides reliable prediction results [53]. The finding that precipitation-based factors are most important for wheat suitability is consistent with other studies that have identified rainfall as a key determinant in marginal production systems [54]. Based on the results from the jackknife gain and AUC tests (Fig 4), it is important to note that the contributions of Bio2, Bio6, Bio9, Bio11, Bio12, Bio16, Bio17, and Bio18 provided the highest gain and AUC tests and provided the determination of soybean planting. Results showed that temperature and precipitation are the most important environmental influences on soybean distribution and expansion potential [1, 55]. A detailed understanding of species distribution is often a prerequisite for species restoration and utilization in ecosystems [56, 57]. Wheat and rice are receiving a lot of attention [58]. These bioclimatic environmental factors have the greatest influence of the land suitability of wheat, soybean, and rice under the current climatic environments and suggest that these aspects themselves contain more valuable information than other variables.

### 3.2. Contribution and importance of the bioclimatic variables under different scenarios

The influence of climatic variables on land suitability for soybean, wheat, and rice were much higher, suggesting that climate change could significantly affect the potential distribution and land suitability of crops. The percent contribution and permutation importance of each bioclimatic variable were obtained using the jackknife tests (Tables 5–7). Ortiz et al. [26] report that future climate will expand wheat area in East Asia. The jackknife test shows that under the current (1970–2000), wheat, Bio1, Bio4 and Bio12 contribute 10.4%, 17.6% and 12.6%, and for future SSP585 (2021–2040), Bio4, Bio13 and Bio19 contribute 17.6%, 11.2% % and 10.5%, while for SSP585 (2041–2060), Bio4, Bio12 and Bio19 contributed 17.3%, 16.3% and 9.6.0% to the MaxEnt model. Climate change may also affect the security of global wheat production in other ways. For example, surface temperatures are projected to increase throughout the 21^st^ century [3], so warming could negatively impact global wheat production [59]. The cumulative percentage contribution of these three variables to wheat under current conditions is 40.6% respectively (Table 5). Bio1, Bio6, and Bio12 had the highest permutation importance percentage analysis under the current and future bioclimatic variables of wheat. Therefore, this suggests that temperature may have a more significant effect on species distribution than precipitation [55].

**Table 5:** Primary contribution percent and permutation importance of the bioclimatic environmental variables impacting on wheat distribution (%) to the simulation results of the MaxEnt model.

Bioclimatic variables	Wheat (Triticum_Aestivum)					
	Current (1970–2000)		SSP585 (2021–2040)		SSP585 (2041–2060)	
	Percent Contribution	Permutation Importance (%)	Percent Contribution	Permutation Importance (%)	Percent Contribution	Permutation Importance (%)
Bio1	**10.4**	**32.4**	8.8	**30.5**	9.0	**30.6**
Bio2	3.4	3.3	3.9	1.3	2.6	0.8
Bio3	7.7	2.5	5.1	1.5	2.3	1.7
Bio4	**17.6**	5.9	**17.6**	4.9	**17.3**	4.1
Bio5	1.7	1.0	5.6	4.2	5.9	3.5
Bio6	2.7	**13.5**	1.9	**13.6**	2.3	**12.7**
Bio7	3.2	0.6	2.1	0.7	4.2	1.9
Bio8	1.7	1.0	2.6	0.3	1.3	0.2
Bio9	9.3	7.3	4.6	11.4	4.6	10.2
Bio10	2.4	0.8	5.1	0.7	6.5	1.2
Bio11	4.1	0.7	4.7	3.8	4.4	2.7
Bio12	**12.6**	**14.2**	7.5	**12.8**	**16.3**	**11.7**
Bio13	6.8	3.2	**11.2**	0.3	1.4	1.0
Bio14	0.3	0.2	0.1	0.0	1.8	0.8
Bio15	1.4	1.2	1.4	0.9	1.8	0.8
Bio16	0.7	0.1	0.1	0.1	0.3	0.1
Bio17	3.3	0.7	5.1	1.5	4.4	1.2
Bio18	0.9	3.8	2.1	8.2	4.2	11.6
Bio19	10.1	3.7	**10.5**	3.3	**9.6**	3.3

Here, SSPs = socio-economic pathways.

Bold: The most influential variable of wheat distribution

For soybean, the current bioclimatic variables Bio3, Bio10 and Bio12 contributed 9.9%, 15.6% and 49.5%; for the future SSP585 (2021–2040) Bio3, Bio10 and Bio12 contributed 11.0%, 14.1% and 50.0%, while for SSP585 (2041–2060) Bio3, Bio10 and Bio12 contributed 13.1%, 11.6% and 51.6% to the MaxEnt, respectively (Table 6). Species distribution depends on environmental gradients and climate variables [60]. We also need the impact of climate change to understand predict potential distribution and future invasion regions for better prevention strategies. The cumulative percentage contribution of these three variables to soybeans is currently 75.0% each. In order to predict land suitability, it is crucial to identify the main environmental variables that have a greater impact on crop land suitability [61]. Bio1, Bio10, and Bio12 had the highest percentile importance analysis for Soybean under current and future bioclimatic variables. Compared with temperature, precipitation is the main determinant and contribution to the rice model. Yearly rainfall is one of the most significant ecological factors that affecting plant growth and development [23]. For rice, current bioclimatic variables such as Bio2, Bio12 and Bio14 contributed 9.4%, 12.9% and 36.0%, and for future SSP585 (2021–2040), Bio12, Bio14 and Bio17 contributed 16.3%, 29.9% and 13.8%, while for SSP585 (2041–2060) the contributions of Bio12, Bio14 and Bio17 to the MaxEnt model were 13.2%, 18.9% and 24.5%, respectively (Table 7). Under current and future bioclimatic variables, Bio9, Bio12, Bio13, Bio17, and Bio18 had the highest percentile importance analysis for rice. Taken together, for rice, the collective contribution of these three bioclimatic variables under current conditions was 58.3%, respectively.

**Table 6:** Primary contribution percent and permutation importance of the bioclimatic environmental variables impacting on soybean distribution (%) to the simulation results of the MaxEnt model.

Bioclimatic variables	Soybean (Glycine_Max)					
	Current (1970–2000)		SSP585 (2021–2040)		SSP585 (2041–2060)	
	Percent Contribution	Permutation Importance (%)	Percent Contribution	Permutation Importance (%)	Percent Contribution	Permutation Importance (%)
Bio1	3.6	**11.0**	3.6	**15.8**	4.3	**16.9**
Bio2	0.1	5.4	3.8	2.4	3.2	3.2
Bio3	**9.9**	1.5	**11.0**	3.0	**13.1**	2.6
Bio4	5.8	5.9	5.5	2.4	3.8	2.5
Bio5	0.5	1.5	0.4	1.7	0.4	0.5
Bio6	1.0	1.1	0.8	0.2	0.4	0.4
Bio7	0.8	1.6	0.4	1.2	0.5	0.4
Bio8	1.5	3.2	2.7	4.5	4.0	5.3
Bio9	6.0	1.1	0.5	4.6	0.6	3.1
Bio10	**15.6**	**12.7**	**14.1**	**10.1**	**11.6**	**6.1**
Bio11	0.6	0.3	0.9	0.1	0.7	0.1
Bio12	**49.5**	**32.8**	**50.0**	**35.9**	**51.6**	**45.7**
Bio13	0.8	1.9	1.3	5.6	1.6	1.2
Bio14	0.2	1.5	0.3	1.1	0.2	0.8
Bio15	2.2	9.0	2.3	4.2	2.0	6.0
Bio16	0.6	6.2	0.4	1.2	0.0	0.3
Bio17	0.3	0.8	0.4	0.5	0.5	1.1
Bio18	0.8	0.3	1.5	3.0	1.2	3.7
Bio19	0.2	2.3	0.2	1.5	0.1	0.3

Here, SSPs = socio-economic pathways.

Bold: The most influential variable for soybean distribution

**Table 7:** Primary contribution percent and permutation importance of the bioclimatic environmental variables impacting on rice distribution (%) to the simulation results of the MaxEnt model.

Bioclimatic variables	Rice (Oryza_Sativa)					
	Current (1970–2000)		SSP585 (2021–2040)		SSP585 (2041–2060)	
	Percent Contribution	Permutation Importance (%)	Percent Contribution	Permutation Importance (%)	Percent Contribution	Permutation Importance (%)
Bio1	3.6	4.7	4.8	5.3	6.1	2.4
Bio2	**9.4**	3.8	7.9	2.6	6.2	1.3
Bio3	1.2	1.1	0.3	0.3	0.8	0.3
Bio4	4.5	1.3	3.0	0.7	3.1	2.6
Bio5	0.9	0.1	1.1	0.5	1.7	0.8
Bio6	1.0	0.5	0.9	2.3	0.5	0.4
Bio7	1.6	3.9	2.0	4.1	1.2	3.5
Bio8	2.6	0.5	3.8	3.4	3.7	2.8
Bio9	4.2	5.6	1.6	**30.6**	1.2	**10.9**
Bio10	3.2	1.5	2.5	0.8	1.7	0.3
Bio11	1.2	1.5	0.6	1.1	0.6	0.9
Bio12	**12.9**	**7.3**	**16.3**	5.4	**13.2**	3.8
Bio13	5.3	**6.1**	3.3	0.9	4.7	3.6
Bio14	**36.0**	1.5	**29.9**	1.3	**18.9**	6.1
Bio15	1.1	0.8	1.3	2.6	1.9	0.7
Bio16	1.5	0.7	2.2	0.7	4.0	0.3
Bio17	8.2	4.7	**13.8**	**9.4**	**24.5**	**30.8**
Bio18	3.4	**51.5**	3.4	**24.1**	3.7	**26.3**
Bio19	1.3	2.9	1.2	4.1	2.5	2.1

Here, SSPs = socio-economic pathways.

Bold: The most influential variable for rice distribution

### 3.3. Potential distribution and change in land suitability under current and future climate

**Fig 6:**
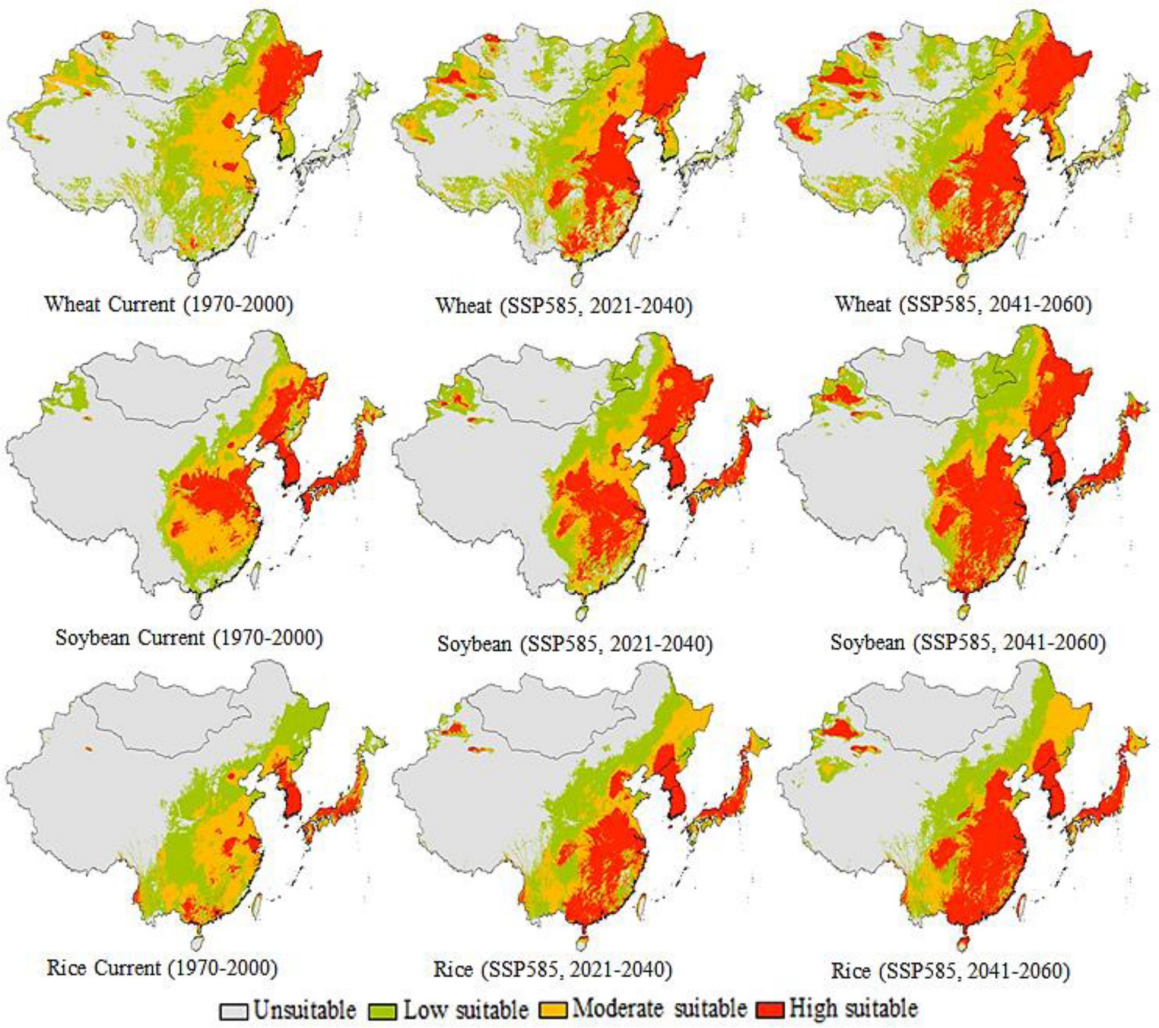
Potential geographic suitable habitat distribution for wheat, soybean and rice production under current (1970–2000) and future (SSP585, 2021–2040; SSP585, 2041–2060) climatic scenarios conditions in East Asia.

The current and future wheat, soybean and rice planting suitability maps predicted by the MaxEnt are presented in Fig 6. The areas marked in red in Fig 6 represent areas where wheat, soybean, and rice are policy impact to be highly suitable for planting, and where crops are actually currently being planted. Although research suggests that wheat area will increase significantly globally by 2035 [30], our finding also suggested that most of these areas are in moderate to high suitability regions. While the yellow areas are expected to be moderately favorable, soybeans are currently unharvested and require more attention for food structure. In addition, the suitability of wheat, soybean, and rice planting in southeastern China, North Korea, South Korea, and Japan showed an increasing trend, while the land suitability in Mongolia and northwestern China showed a downward trend. The rapid development within the soybean industry in North and South America has made them the largest soybean export base for the world [57]. East Asia has become a big soybean exporter as soybean production has increased in size and level in recent decades [62]. Overall, climate change is projected to increase the suitability of East Asian lands for wheat, soybean, and rice. Other areas showing less potential in gray areas and unsuitable for growing wheat, soybeans and rice include Mongolia in northwestern China. These results are similar to previous studies on the spatial distribution characteristics of soybean climate suitability in northeast China [63, 64]. Mongolia [65], identified as the most suitable soybean growing area, is consistent with our suitable habitat area. Under the SSP585 2021–2060 scenario, it is expected that the high-suitable planting area of wheat, soybean and rice will continue to increase significantly in the future, however, both moderate and low suitability areas are projected to decrease. In the future SSP585 2041–2060 scenario, the unsuitable planting area of wheat, soybean and rice will be slightly reduced. In recent years, China’s annual soybean consumption is about 110 million tones, and annual soybean imports are about 90 million tones, accounting for more than 60% of global trade of soybean [64]. Compared to the SSP585 2021–2040 scenario, the same trend but larger changes are expected for the SSP585 2041–2060 scenarios.

The area ratios for the various conformance classes are shown in Table 8. Compared to the current period, the total area projected for the SSP585 2021–2040 and SSP585 2041–2060 scenarios have changed significantly. However, the suitability of the land for wheat cultivation has changed significantly. The MaxEnt also accurately predicted potential rice, wheat, and soybean acres both nationally and globally [19]. Accurate predictions were achieved in part due to the highest analytic power of the MaxEnt [36, 55]. Also, the prediction accuracy of land suitability is greatly affected by the number of occurrence points [66]. The total high suitability area of wheat under the SSP585 2021–2040 and SSP585 2041–2060 scenarios are projected to increase significantly by 66.1% and 75.1%, respectively, compared with the current one. However, in the SSP585 2021–2040 and SSP585 2041–2060 scenarios, the total moderately suitable area is projected to increase slightly by 0.1% and 6.4% from the current level, respectively. Our findings are consistent with those of Qian et al. [67], reported that the land unsuitable for wheat area will decrease by 2050. In addition, total unsuitable area is projected to decrease by -54.5% and -98.5%, and marginal suitable area will decrease by -3.4%, and by -11.4% in the SSP585 2021–2040 and SSP585 2041–2060 scenarios compared to the current scenario. Earlier work has suggested that climate change may raise wheat yield in high-latitude while causing greater losses in warmer regions [61]. Elsgaard et al. [68] reported that future climate would expand wheat areas in East Asia. Total unsuitable and moderately suitable soybean areas are projected to decrease significantly in future scenarios, respectively, compared to the current situation. In the SSP585 2021–2040 and SSP585 2041–2060 scenarios, the total soybean high suitability area is expected to increase significantly by 58.9% and 68.5%, respectively, compared with the current level. The impact of land-use transition will increase the spatial extent of unsuitable habitats beyond what was predicted [69]. The total area of unsuitable, low-suitable and moderately suitable rice in the future scenarios is expected to be significantly reduced compared with the current situation. Ortiz et al. [26] also reported that future climate will improve high land suitability for rice production. However, in the SSP585 2021–2040 and SSP585 2041–2060 scenarios, the total high-suitability rice area is projected to increase significantly by 73.1% and 81.9%, respectively, from the current level. Climate variables related to precipitation are important to ensure flowering and maturation of rice crops [70]. These finding show that the high suitable regions improved significantly in the long run, while the total number of suitable regions decreased. This is because the areas of poor, low, and medium fit are much larger than the areas of high fit (Fig 6).

**Table 8:** Changes in land suitability for wheat, soybean and rice under the current and future (SSP585; 2021–2040; 2021–2060) climatic conditions.

Suitability Index	Climate scenarios	Time period	Wheat ×10^5^ km^2^	Change of area (%)	Soybean ×10^5^ km^2^	Change of area (%)	Rice ×10^5^ km^2^	Change of area (%)
Unsuitable	Current	1970–2000	9.73		16.94		16.97	
	SSP585	2021–2040	6.30	-54.5	13.48	-25.7	14.73	-15.3
	SSP585	2041–2060	4.90	-98.5	12.00	-41.2	12.45	-36.3
Low suitable	Current	1970–2000	11.09		4.12		6.14	
	SSP585	2021–2040	10.73	-3.4	5.09	19.1	5.28	-16.3
	SSP585	2041–2060	9.95	-11.4	4.89	15.8	6.07	-1.2
Moderate suitable	Current	1970–2000	6.00		5.13		4.44	
	SSP585	2021–2040	6.01	0.1	3.91	-31.3	4.24	-4.9
	SSP585	2041–2060	6.42	6.4	3.66	-40.2	3.53	-25.7
High suitable	Current	1970–2000	1.94		2.59		1.22	
	SSP585	2021–2040	5.73	66.1	6.30	58.9	4.53	73.1
	SSP585	2041–2060	7.80	75.1	8.22	68.5	6.72	81.9

### 3.4. Determination of the dominant environmental factors

**Fig 7:**
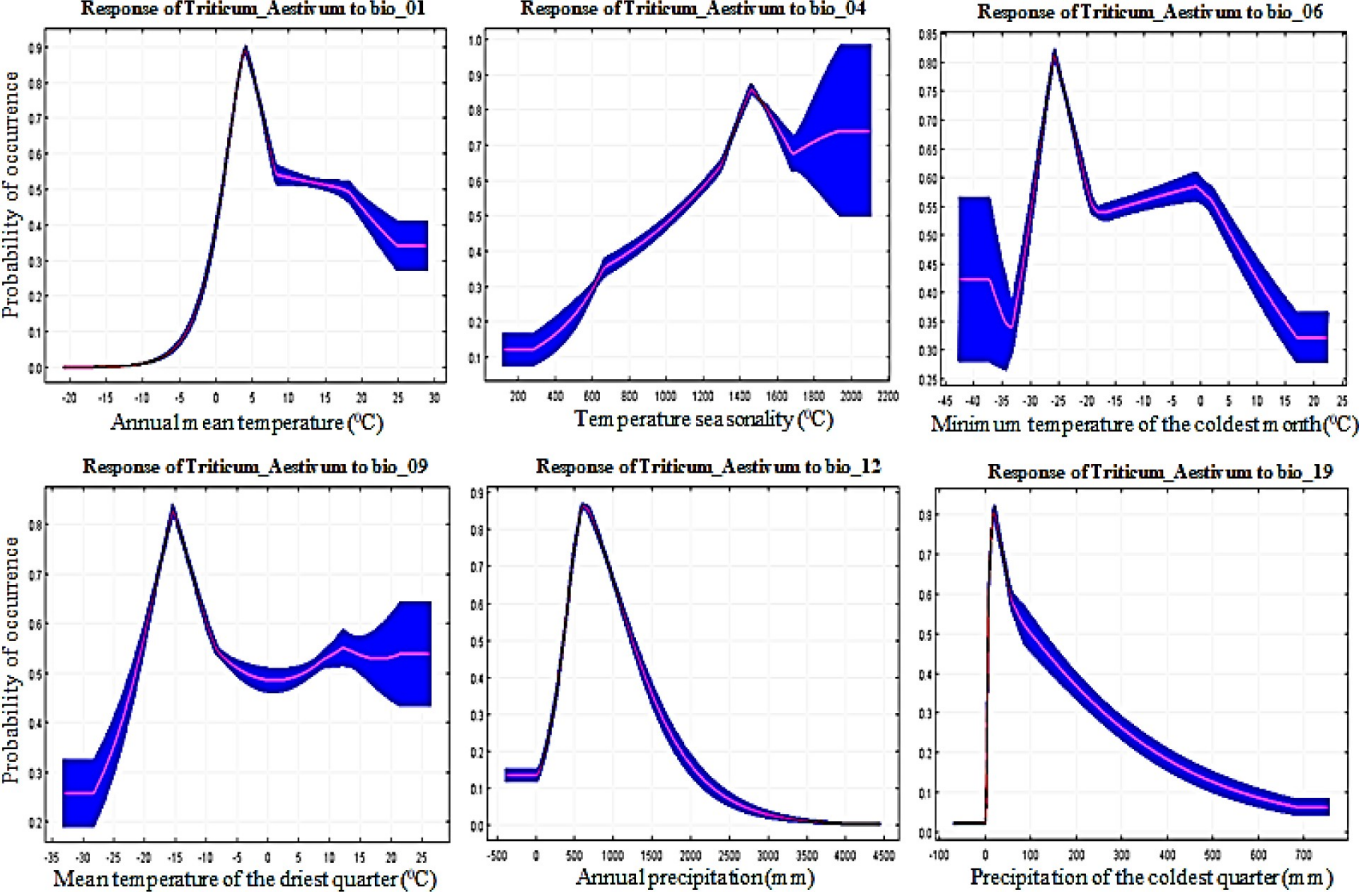
Wheat (*Triticum_Aestivum*) response curves derived from MaxEnt model showing the influence of bioclimatic environmental variables; Bio_01, Bio_04, Bio_06, Bio_09, Bio_12, and Bio_19 in East Asia.

**Fig 8:**
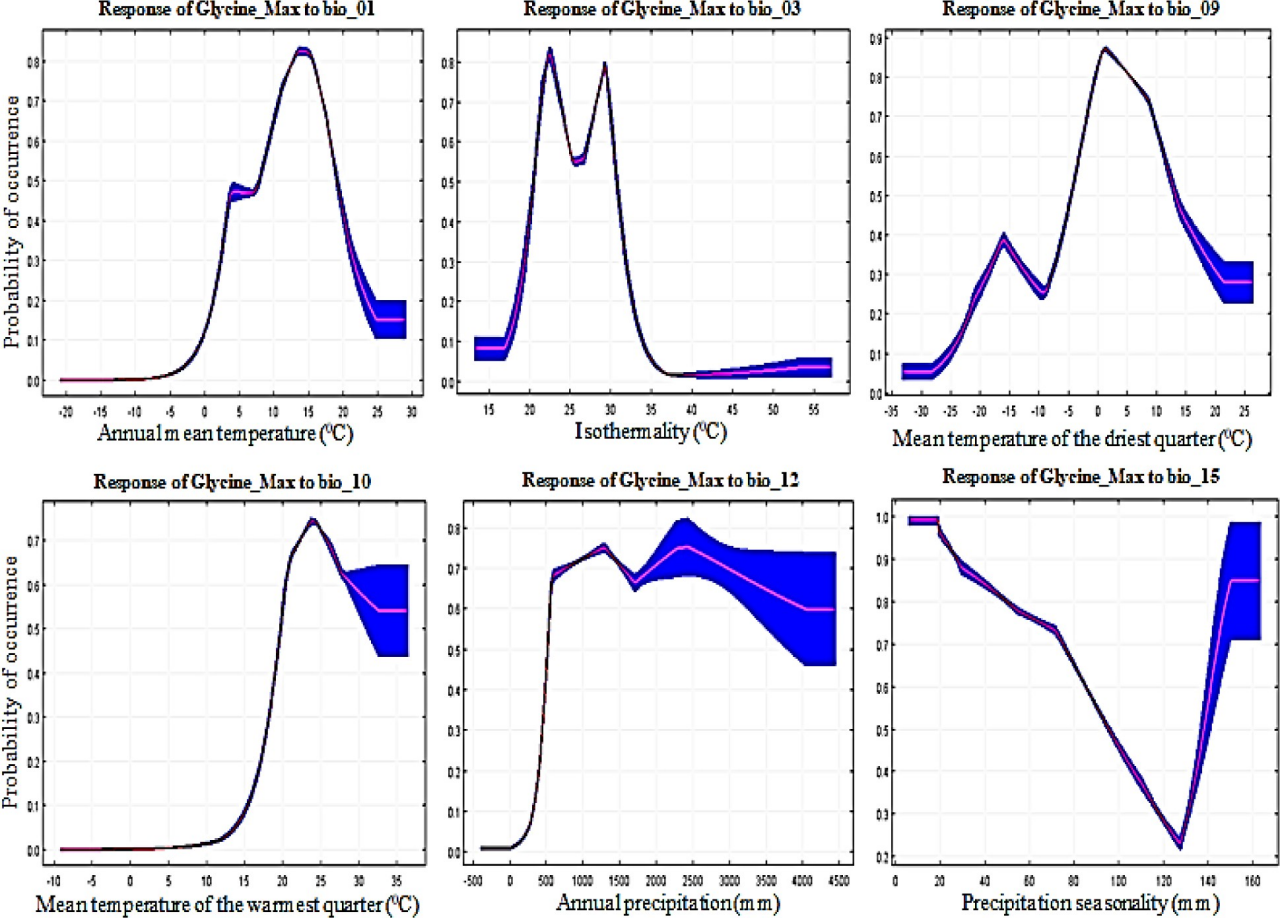
Soybean (*Glycine_Max*) response curves derived from MaxEnt model showing the influence of bioclimatic environmental variables; Bio_01, Bio_03, Bio_09, Bio_10, Bio_12, and Bio_15 in East Asia.

**Fig 9:**
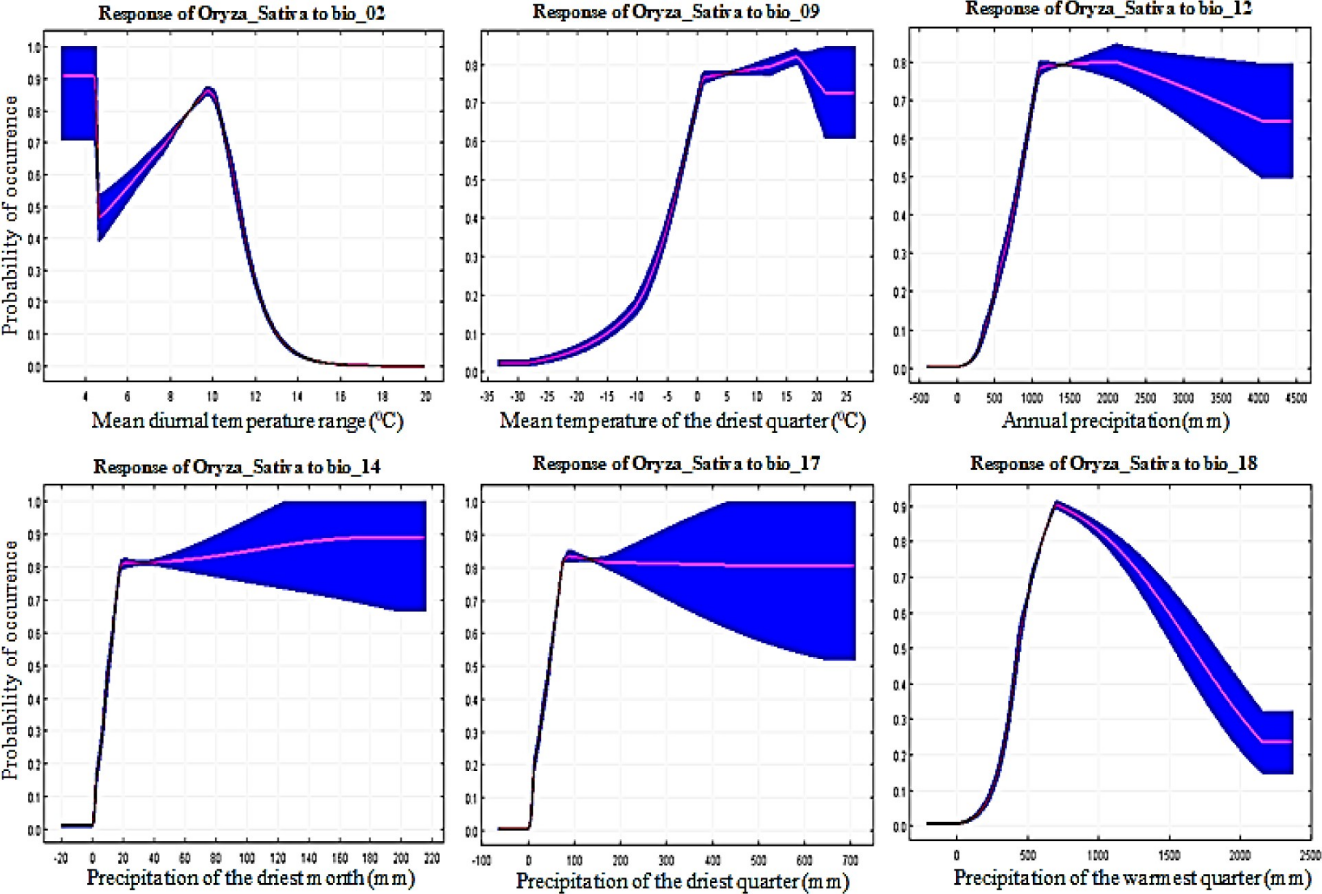
Rice (*Oryza_Sativa*) response curves derived from MaxEnt model showing the influence of bioclimatic environmental variables; Bio_02, Bio_09, Bio_12, Bio_14, Bio_17, and Bio_18 in East Asia.

To predict land suitability, it is important to identify key environmental variables that have a greater influence on cropland suitability [61]. The cumulative contribution of these six environmental variables was high at 62.7%, proving that these six factors play an important role in the potential distribution of wheat. From the response curves (Fig 7), we obtained Bio1 for the main bioclimatic variables as -5 to 30°C; Bio4, 200 to 1400°C; Bio6, -35 to -20°C; Bio9, -30 to -10°C°C; Bio12, 400 to 3500 mm; and Bio19, 50 to 700 mm. There is little work using regional climate models to study crop suitability in East Asia; however, it is also a valid source for study regional crop suitability model [71]. Soybean curves show the association between bioclimatic variables and soybean presence probabilities. According to the soybean response curve obtained, soybeans prefer Bio1 at 5 to 25°C; Bio3, at 20 to 35°C; Bio9, at -10 to 20°C; Bio10, at 15 to 35°C; Bio12, at 500 to 4500 mm; and Bio15, 140 to 160 mm (Fig 8). The cumulative contribution rate of these six bioclimatic variables was high at 86.8%, proving that these six factors play an important role in the potential distribution of soybeans. One study suggested that soybean yields could decline by 16% depending on future summer warming [72]. According to obtained rice response curves, rice prefers Bio2 at 4 to 14°C; Bio9, -5 to 25°C; Bio12, 600 to 4200 mm; Bio14, 20 to 220 mm; Bio17, 80 to 700 mm; and Bio18, 500 to 2100 mm (Fig 9). This result is consistent with previous studies showing that changes in temperature and daytime can lead to changes in rice distribution [72, 73]. The cumulative contribution rate of these six bioclimatic variables was high at 74.1%, proving that these six factors play an important role in the potential distribution of rice. Thus, this suggests that temperature may have a greater effect on rice distribution than precipitation [59].

## 4. Conclusion

We successfully developed a MaxEnt model that predicted the potential distribution of current and future land suitable for wheat, soybean, and rice cultivation. The accuracy of the MaxEnt model is excellent, with mean AUC values ranging from 0.833 to 0.882 for all models evaluated. The jackknife test showed that for wheat, Bio4 and Bio12 contributed 17.6% and 12.6%, for soybean, Bio10 and Bio12 contributed 15.6% and 49.5%, and for rice, Bio12 and Bio14 contributed 12.9% and 36.0%. In addition, the suitability of wheat, soybean, and rice planting in southeastern China, North Korea, South Korea, and Japan showed an increasing trend, while the land suitability in Mongolia and northwestern China showed a downward trend. The climate change is projected to improved land suitability for soybean, wheat, and rice in East Asia. The simulation results showed that the unsuitable area of wheat, soybean and rice would decrease by -98.5%, -41.2% and -36.3% on average in 2060 compared with the current land suitability. In contrast, by 2060, the highly suitable planting area of wheat, soybean and rice is projected to increase by 75.1%, 68.5% and 81.9%, respectively, compared with the current land suitability. In light of our findings, it is our recommendation that further analysis is needed to identify land use changes and determine the effective area of suitable lands that can be targeted for wheat, soybean and rice cultivation in order to ensure sustainable production and mitigate food insecurity. This study should be used as a proof of concept to demonstrate an approach to describe suitable crops areas under current and future bioclimatic variables and establish policies on East Asia geography and climate situation.
